# A Mixed Methods Analysis of Medical Needs Following the Noto Peninsula Earthquake: Bridging the Gap Between Digital Narratives and Disaster Policy

**DOI:** 10.7759/cureus.103555

**Published:** 2026-02-13

**Authors:** Takao Sakai

**Affiliations:** 1 Orthopedics, Nagoya Kyoritsu Byoin, Nagoya, JPN

**Keywords:** disaster medicine, disaster planning, noto peninsula earthquake, primary health care, professional burnout, qualitative analysis, social media

## Abstract

Introduction

The chronological shift in medical needs following major seismic events, particularly the exhaustion of local responders, remains under-researched. While acute physical damage is often well documented, the subacute phase presents unique challenges that are difficult to capture through traditional surveillance.

Objective

This study aimed to analyze medical needs operationally defined as thematic content derived from professional narratives, following the Noto Peninsula Earthquake, and to triangulate these qualitative insights with temporal trends in digital discourse by conducting a mixed-methods analysis of digital narratives found in YouTube videos.

Methods

An exploratory sequential mixed-methods design was used. In the first (qualitative) phase, thematic analysis was conducted on 12 YouTube videos featuring medical professionals to extract in-depth experiences using MAXQDA 2024 (VERBI Software, Berlin, Germany). In the second (quantitative) phase, to examine the temporal prevalence of the identified themes, a trend analysis was performed on 300 videos uploaded between January 1 and December 31, 2024. Using the YouTube Data API, data were collected chronologically to visualize the monthly distribution of needs.

Results

The qualitative analysis identified three themes: (1) acute material shortages, (2) mid-to long-term shelter hygiene/mental health, and (3) exhaustion of local staff. The quantitative triangulation revealed a distinct temporal lag: while infrastructure-related content peaked in January (20 videos), content related to "Staff Support" (theme three) peaked in February (38 videos), one month after the earthquake.

Conclusion

This study quantitatively triangulated the "February Peak," a phenomenon in which local staff burnout peaks exactly when external support begins to wane. Future disaster plans must include explicit protocols for mandatory rest and the deployment of relief teams targeting the subacute phase (one to two months post-disaster).

## Introduction

The magnitude 7.6 Noto Peninsula earthquake that occurred on January 1, 2024, caused severe damage to a wide area of the Noto Peninsula and reaffirmed the importance of disaster prevention plans in the medical care system [1]. The damage was exacerbated by the tsunami, landslides that destroyed roads, and collapsed houses, making post-earthquake recovery difficult [2]. In areas with large elderly populations, issues such as increased medical needs, resource constraints, and health problems in evacuation centers have been identified.

Disaster medicine often prioritizes immediate crisis responses over research activities, resulting in a scarcity of documented lessons learned by medical professionals during the acute phase. However, these insights are crucial for future improvements. To address this gap, this study adopted a mixed-methods approach, combining the depth of qualitative narratives with the breadth of quantitative trend analysis using digital data. Specifically, the objective was to identify 'medical needs' as expressed in professional narratives and to triangulate their temporal distribution by conducting a mixed-methods analysis of digital narratives found in YouTube videos in digital discourse. This approach aims to bridge the gap between static disaster prevention plans and the dynamic realities experienced by medical professionals on the ground.

## Materials and methods

Study design

This study used an exploratory sequential mixed-methods design. The first phase involved a qualitative thematic analysis of the selected narratives to identify key themes. The second phase involved a quantitative trend analysis of a larger dataset to triangulate the temporal distribution of themes with online discourse trends.

Phase one: qualitative data collection and analysis

Data collection focused on YouTube (Google LLC, California, USA) videos uploaded between January 1, 2024, and December 31, 2024. [3] The inclusion criteria were as follows: (1) the title must contain "Noto Peninsula Earthquake" and "medical care,” and (2) the video must include a narrative by a medical professional detailing their experience in the disaster-affected areas. Videos were excluded if the content was unrelated to medical care or not based on direct experience.

To ensure the exhaustiveness and accuracy of the data extraction, this study employed a " human-in-the-loop " (HITL) approach. While the subtitle text was initially generated using YouTube's AI-based automatic captioning function, all transcripts underwent manual verification by researchers to correct errors and ensure contextual precision before being imported into MAXQDA 2024 (VERBI Software, Berlin, Germany) for analysis [4]. The analysis continued until theoretical saturation was reached, defined as the stage at which the integration of new data ceased to yield additional insights or refine the properties of established themes.

Phase two: quantitative triangulation strategy

To examine the temporal alignment of the themes extracted from the qualitative phase with broader digital discourse, a quantitative trend analysis was conducted using the YouTube Data API v3 (Google, Mountain View, California, USA).

Data Collection

To minimize selection bias and ensure reproducibility, data collection was performed systematically using the YouTube Data API v3. The search query was defined as 'Noto Peninsula Earthquake' AND 'Medical' (Japanese: Noto-hanto Jishin and Iryo). The search parameters were set to retrieve videos published between January 1, 2024, and December 31, 2024, sorted by publication date (order='date') to capture chronological progression throughout the year. The top 300 videos were extracted for the analyses.

Keyword Analysis

A controlled vocabulary was developed based on the three themes identified in Phase 1. We defined specific Japanese search keywords for each category to detect relevant content within the video metadata (title and description).

Theme one (acute Infrastructure): "Road" (Doro), "Water outage" (Dansui), "Communication" (Tsushin), "Helicopter" (Heli), "Disaster Medical Assistance Teams (DMAT)", "Isolation" (Koritsu), "Rescue" (Kyujo).

Theme two (shelter life): "Evacuation center" (Hinan-jo), "Infection" (Kansen), "Nutrition" (Eiyo), "Mental" (Mental), "Pneumonia" (Haien), "Oral care" (Kokuu), "Toilet" (Toilet).

Theme three (staff support): "Medical worker" (Iryo-jujisha), "Nurse" (Kangoshi), "Doctor" (Ishi), "Exhaustion" (Hihei), "Rest" (Kyusoku), "Supporter" (Shiensha), "Long-term" (Choki), "Dispatch" (Haken).

Data Processing

Data processing was performed using Python (version 3.10) and the pandas library. For each video, the title and description were concatenated into a single text string. A Boolean search was applied; if a video contained at least one keyword from a specific theme, it was flagged as relevant to that theme. The monthly frequency of videos for each theme was calculated to visualize temporal shifts in social interest and medical needs.

Ethical considerations

This study analyzed videos publicly available on YouTube. According to the ethical guidelines for medical and health research involving human subjects in Japan, research utilizing exclusively publicly available information is exempt from mandatory institutional ethical review. According to the ethical guidelines of Nagoya Kyoritsu Hospital, this study was exempt from formal review. To prioritize scientific reproducibility, the dataset was not anonymized. A complete list of the analyzed videos is provided in the Appendix.

## Results

Phase one: qualitative themes

Theoretical saturation was reached after analyzing 12 videos. In total, 48 initial codes were generated using MAXQDA24. Through iterative analysis, these codes were categorized into three distinct themes representing the chronological and structural progression of medical needs: (1) acute phase material and information disruption, (2) subacute phase shelter health management, and (3) long-term sustainability of the medical workforce (Table 1).

**Table 1:** Qualitative Themes and Representative Codes Identified from Narrative Analysis The table summarizes the three distinct themes extracted from the thematic analysis of the video narratives (n=12). "Focus Area" indicates the temporal and situational context of the medical needs. "Typical Codes" lists the primary tags generated during qualitative coding using MAXQDA24.

Theme	Focus Area	Typical codes	
Theme One	Acute Phase (Physical)	Infrastructure collapse; Water shortages; Food shortages; Road passage difficulty; Loss of hospital communication means; Direct arrival of emergency patients; Hospital water outages; Difficulty in grasping the number of victims; Weather affecting wide-area helicopter transport	
Theme Two	Sub-acute Phase (Shelter)	Spread of infectious diseases; Insufficient ventilation in shelters in winter; Worsening of condition in patients with underlying diseases; Prevention of disaster-related deaths; Burden of snow removal work; Need for mental care in evacuation shelters; Anxiety of disaster victims; Bathing difficulties for those requiring care; Loss of health insurance cards; Relief measures for lost cards; Mental stress	
Theme Three	Sub-acute Phase (Staff)	Lack of rest for medical workers; Sense of obligation of medical workers in disaster areas; Mental support for medical workers; Need for public support for clinics; Medical and nursing staff exhaustion; Sleep deprivation of medical workers; Continuous duty; Beliefs of doctors in disaster areas; Situational adaptability;	

Theme One

Material shortages and information confusion due to the earthquake (acute phase). This theme captures the immediate paralysis of medical functions caused by physical infrastructure failure. The most frequently observed codes were "infrastructure collapse" and "road passage difficulty". A critical finding in this phase was the "loss of hospital communication means". Qualitative analysis revealed that this was not merely a loss of the Internet but a total disruption of fixed telephone lines, rendering hospitals unable to contact ambulance crews or confirm the acceptance of severely ill patients. Consequently, hospitals faced the "direct arrival of emergency patients" without prior notification, leading to chaotic acceptance preparations. Furthermore, environmental factors such as "hospital water outages" and "weather affecting wide-area helicopter transport" severely limited the capacity to transfer critical patients to higher-level facilities.

Theme Two

Support for victims in evacuation shelters (sub-acute phase). As the disaster response shifted to the sub-acute phase, the focus of the codes moved to public health management within crowded living spaces. The highest frequency codes in this category were related to the "spread of infectious diseases,” exacerbated by "insufficient ventilation in shelters in winter.” Vulnerable populations faced specific risks, indicated by the codes "worsening of condition in patients with underlying diseases" and "bathing difficulties for those requiring care.” Social determinants of health also emerged as a priority, with distinct codes identifying administrative hurdles such as "loss of health insurance cards" and the need for "relief measures for lost cards". The narrative data highlighted that the medical crisis in this phase was driven by the "mental stress" of prolonged evacuation and the lack of hygiene resources such as water and food.

Theme Three 

Support for medical workers in disaster areas (sub-acute to chronic phase). The final theme highlights the fragility of the human support system. While external teams provided aid, local "medical and nursing staff exhaustion" became a dominant concern. Codes such as "sleep deprivation of medical workers" and "continuous duty" pointed to a system sustaining itself solely on the "beliefs of doctors in disaster areas" rather than adequate staffing. Crucially, the analysis identified a specific need for "mental support for medical workers,” distinct from general victim support. Despite these pressures, positive adaptive behaviors were noted, with codes for "situational adaptability" describing the ability of staff to flexibly respond to changing needs, and "gratitude for support from others" serving as a protective psychological factor.

Phase two: quantitative triangulation

To triangulate the qualitative findings with temporal trends in digital discourse, a quantitative trend analysis was conducted on a dataset of 300 videos (January 1-December 31, 2024). The chronological distribution of videos related to each theme is presented in Table 2.

**Table 2:** Monthly Trends of Video Counts by Theme (n = 300) The table displays the monthly frequency of videos containing keywords related to each theme. Theme one (acute) peaked immediately, whereas Theme three (staff support) showed a delayed peak in February and a resurgence in December. Because a single video may contain keywords belonging to multiple themes, the sum of the percentages across themes may exceed 100%. Keyword counts reflect the number of videos containing at least one keyword from a specific category.

Month (2024)	Theme One: Acute/Infrastructure	Theme Two: Shelter Life	Theme Three: Staff Support
January	20	1	1
February	2	13	38
March	1	2	15
April	0	1	10
May	0	0	8
June	0	0	6
July	0	0	5
August	0	0	5
September	1	0	5
October	0	0	4
November	0	0	4
December	0	0	19

Immediate but Transient Focus on Infrastructure (Theme One)

Videos related to Theme one (acute/infrastructure) peaked in January (n=20). This reflects the intense initial focus on physical damage, logistics, and DMAT deployment. However, the frequency of this theme declined rapidly in the subsequent months. This suggests that infrastructure issues were perceived as having ‘declined' relatively quickly in the digital discourse.

Rapid Decline of Shelter Attention (Theme Two)

Theme two (shelter life) peaked in February (13 videos), corresponding to the prolongation of evacuation life. However, the content related to this theme decreased sharply from March. This suggests a potential discrepancy: while the actual need for hygiene and mental health support in shelters likely persisted or worsened over time, public or social media attention to these chronic issues faded relatively quickly compared to the enduring narrative of staff struggle.

The "Time Lag" of Medical Staff Burnout (Theme Three)

In contrast to infrastructure, Theme three (staff support) showed a distinct temporal lag. The frequency of videos on staff exhaustion did not peak in January but reached its maximum in February (38 videos), surpassing infrastructure-related content. This quantitative finding triangulates the qualitative insight that the crisis for local responders deepens in the sub-acute phase, exactly when the initial adrenaline fades, and external support begins to withdraw. Furthermore, unlike the other themes, Theme three maintained a consistent presence throughout the year and exhibited a significant resurgence in December (19 videos). This resurgence reflects retrospective media coverage and the persistent and long-term burden on local healthcare providers.

## Discussion

Qualitative analysis revealed that medical needs shifted from acute material shortages to mid-to long-term challenges involving evacuation shelter hygiene and the exhaustion of medical professionals. By integrating these findings with quantitative triangulation and the existing literature, significant lessons can be derived regarding the gap between current disaster planning and reality.

The "time lag" of medical staff exhaustion

The most significant finding from this mixed-methods analysis is the quantitative triangulation of the "February Peak." The qualitative narratives revealed that local staff worked through the acute phase, driven by a "sense of obligation," but faced severe burnout as the phase transitioned. This insight was powerfully corroborated by the quantitative data, which showed a clear one-month lag between the peak of infrastructure needs (January) and the peak of staff support needs (February). This aligns with reports from the closest university hospital, which faced a chaotic patient influx immediately post-disaster [5]. However, the data add a critical temporal dimension to this staff exhaustion. This suggests a structural vulnerability: external support often targets the acute phase, but the need for staff relief peaks in the sub-acute phase when attention begins to wane.

Challenges in evacuation shelters and the "silence" of chronic needs

The video analysis highlighted "Infectious disease epidemics" and "Worsening of chronic illnesses.” This aligns with the literature, which reports that delays in infrastructure restoration lead to poor ventilation and hygiene issues [5]. Furthermore, the "worsening of physical condition" noted in videos is consistent with studies finding shelter meals deficient in energy and protein [6] and lacking essential fatty acids [7]. The "Mental stress" observed in the videos is also corroborated by studies indicating increased risks of postpartum depression [8] and worsening psychiatric symptoms in disaster-affected areas [9]. However, the quantitative data showed a sharp decline in shelter-related videos after March, suggesting that while clinical needs persisted, social attention faded. This "silence" in the digital narrative may mask ongoing chronic health issues in the recovery phase.

Gap analysis: discrepancies between disaster plans and reality

A review of the Wajima City Regional Disaster Prevention Plan revealed critical gaps compared to the findings of this study. The plan was reviewed by cross-referencing specific protocols against the three primary themes identified in the qualitative phase to identify functional gaps between administrative intent and field reality. First, regarding isolation measures, although the plan relies on helicopter transport [10], the videos highlighted that weather conditions rendered these ineffective, a reality often underestimated in paper-based plans. Second, regarding support for local staff, the plan lacks specific provisions for the rest and mental support of local medical personnel. Systemic challenges in certifying disaster-related deaths [11] further complicate the long-term recovery process. The findings strongly suggest that relief for local responders should be codified in disaster response protocols. Finally, regarding medicine supply, reliance on self-stockpiling [12] proved insufficient. Specific vulnerabilities, such as interruptions in chemotherapy [13] and muscle mass loss in nursing home residents [14], highlight the need for sustained rather than just acute medical logistics.

Strategic role of private clinics in disaster primary care

Furthermore, this study highlights the urgent need to integrate private clinics into government disaster prevention frameworks, both physically and informationally. Geographically, the population within an evacuation shelter largely overlaps with the patient catchment area of the local private clinics. Consequently, local private practitioners ("Kakaritsuke-i") possess pre-disaster knowledge of the evacuees' medical histories, which is crucial for managing chronic conditions that constitute the majority of medical needs in the subacute phase [15]. Unlike external relief teams (e.g., DMAT), which rotate frequently, local clinics provide the continuity of care required to address these long-term challenges.

However, current government support is heavily skewed toward designated disaster-based hospitals. Chan et al. emphasized that "inadequate communication" and "misinformation" are critical barriers in mass-casualty incidents, advocating the use of advanced IT systems to manage resources in chaotic environments [16]. However, the findings imply that this "digital transformation" has not reached the grassroots level. Private clinics often operate in information silos, cut off from regional command centers. To prevent disaster-related deaths in aging societies, the government must officially recognize private clinics as Disaster Primary Care Hubs. This involves investing in their physical resilience (water/power) and, crucially, establishing a secured wireless communication network that connects these clinics to the central disaster response system, enabling real-time data sharing on patient needs and resource shortages, as envisioned by the authors decades ago.

Methodological reflection and limitations

The interpretation of the results is subject to several limitations. First, because the analysis was limited to YouTube videos, there is an inherent selection bias; the data reflect only the voices of those who chose to disseminate information digitally. Specifically, the findings likely exclude the perspectives of the most overwhelmed personnel who lack the time or mental energy to engage in online activities. Therefore, the exhaustion observed in this study may underestimate the severity of reality. Second, while the AI-based captioning was verified by researchers, minor inaccuracies in transcription cannot be entirely ruled out. Third, the quantitative trends reflect social interest or volume of discourse on YouTube, which serves as a proxy for actual medical needs but does not directly measure clinical epidemiology. Finally, this study focused on the 2024 Noto Peninsula Earthquake, and the generalizability of the 'one-month lag' in staff burnout to other disaster settings requires further verification in future studies.

## Conclusions

This study clarifies the discrepancies between disaster plans and reality following the Noto Peninsula Earthquake. The primary contribution is the quantitative triangulation of the "February Peak" in staff exhaustion, demonstrating that a rigorous analysis of digital narratives can uncover vital lessons often overlooked by traditional surveillance. This highlights the critical value of open-source data as a feedback mechanism for future plans. Consequently, these insights highlight the potential for disaster management frameworks to consider dedicated support for local responders during the subacute phase.

## Appendices

**Table 3:** List of YouTube Videos Analyzed in the Qualitative Analysis

Video No.	Video Title (English Translation)	URL
1	"Medical Do-Naru!" (Jan 12): The Scene of Disaster Medicine in the Noto Peninsula Earthquake	https://www.youtube.com/watch?v=C1FoijUMwrk
2	"Overwhelming Shortage of Medical Manpower": A Doctor Rushing to the Disaster Area [Ishikawa/Noto Peninsula Earthquake]	https://www.youtube.com/watch?v=XIRnXCI0OFU
3	[Noto Peninsula Earthquake] What Should People Requiring Medical Care Do?	https://www.youtube.com/watch?v=7c6Bo9Fts-U
4	"Seismic Intensity 7" Noto Peninsula Earthquake: Local Medical Staff Also Victims - Sapporo DMAT Sees Reality: "Nothing Goes According to Plan"	https://www.youtube.com/watch?v=fFqU-90GSLo
5	[Expert Explanation/Noto Peninsula Earthquake] Medical Situation in Evacuation Centers and Points for Future Evacuation Life	https://www.youtube.com/watch?v=ahvcLcEbHng
6	"Medical Needs in Disaster Areas Change Daily": AMDA Activity Report One Month After the Noto Peninsula Earthquake	https://www.youtube.com/watch?v=mtNdckuoons
7	[Noto Peninsula Earthquake] Day 5: "Barely Surviving" by First Relief Supplies - Situation at the Medical Scene	https://www.youtube.com/watch?v=2qZr4C2Gsnc
8	[Noto Peninsula Earthquake] Foot Care for Victims: The Current Status of Disaster Medicine Felt by the Osaka Medical Team	https://www.youtube.com/watch?v=EHttG1PQIIk
9	Serious Medical System Crisis and Material Shortage in Disaster Areas	https://www.youtube.com/watch?v=S7XdrSQUCiY
10	2024 Noto Peninsula Earthquake: Reconstruction of Family Doctor's Medical Care	https://www.youtube.com/watch?v=muaeMOE0cFc
11	Special Talk: JMA President Matsumoto & Ishikawa Medical Association President Yasuda - About Medical Care in the Noto Peninsula Earthquake Disaster Areas	https://www.youtube.com/watch?v=Kdq5hVcScNw
12	[Noto Peninsula Earthquake] Protecting Regional Medicine: Three Days at a Damaged Hospital Amidst Ceiling Collapse and Water Outage	https://www.youtube.com/watch?v=2dwJqC8FadA
